# Performance of Lipoarabinomannan Assay using Cerebrospinal fluid for the diagnosis of Tuberculous meningitis among HIV patients

**DOI:** 10.12688/wellcomeopenres.15389.2

**Published:** 2019-09-30

**Authors:** Richard Kwizera, Fiona V. Cresswell, Gerald Mugumya, Micheal Okirwoth, Enock Kagimu, Ananta S. Bangdiwala, Darlisha A. Williams, Joshua Rhein, David R. Boulware, David B. Meya

**Affiliations:** 1 Infectious Diseases Institute, College of Health Sciences, Makerere University, Kampala, Uganda; 2 Clinical Research Department, London School of Hygiene and Tropical Medicine, London, WC1E 7HT, UK; 3 MRC-UVRI and LSHTM Uganda Research Unit, Entebbe, Uganda; 4 Department of Medicine, School of Medicine, College of Health Sciences, Makerere University, Kampala, Uganda; 5 Division of Infectious Diseases and International Medicine, Department of Medicine, University of Minnesota, Minneapolis, MN, 55455, USA

**Keywords:** Tuberculous meningitis, extra-pulmonary TB, lipoarabinomannan, TB-LAM, Xpert MTB/Rif Ultra, HIV, Diagnostics, cerebrospinal fluid

## Abstract

**Background:** The diagnostic utility of the *Mycobacteria tuberculosis* lipoarabinomannan (TB-LAM) antigen lateral flow assay on cerebrospinal fluid (CSF) for the diagnosis of tuberculous meningitis (TBM) has not been extensively studied and the few published studies have conflicting results.

**Methods:** Lumbar CSF from 59 HIV-positive patients with suspected TBM was tested with TB-LAM and Xpert MTB/Rif Ultra. The diagnostic performance of CSF TB-LAM was compared to positive CSF Xpert MTB/Rif Ultra (definite TBM) and a composite reference of probable or definite TBM according to the uniform case definition.

**Results:** Of 59 subjects, 12 (20%) had definite TBM and five (9%) had probable TBM. With reference to definite TBM, CSF TB-LAM assay had a diagnostic sensitivity of 33% and specificity of 96%. When compared to a composite reference of definite or probable TBM, the sensitivity was 24% and specificity was 95%. There were two false positive tests with TB-LAM (3+ grade). In-hospital mortality in CSF TB-LAM positive patients was 17% compared to 0% in those with definite TBM by Xpert MTB/Rif Ultra but negative LAM.

**Conclusions:** Lumbar CSF TB-LAM has a poor performance in diagnosing TBM. Both urine TB-LAM and Xpert Ultra should be further investigated in the diagnosis of TBM.

## Introduction

In many human immunodeficiency virus (HIV) endemic countries, tuberculous meningitis (TBM) is the second most common cause of adult meningitis after cryptococcal meningitis ^1^, and accounts for 1–5% of all tuberculosis (TB) cases ^2^. TBM is the most severe form of TB and causes substantial morbidity and mortality in children and immunocompromised adults ^3, 4^. HIV infection is known to increase the risk of death in patients with TBM, as does TBM stage at the time of treatment initiation ^2, 5^. As is the case in cryptococcosis, high-quality nursing care is a critical component in managing TBM patients ^6^.

Similarly, diagnosis of TBM is very challenging, especially in resource-limited settings where diagnosis relies on a combination of clinical, radiological and laboratory findings. The World Health Organisation (WHO) recommends Xpert MTB/RIF Ultra for the diagnosis of TBM using cerebrospinal fluid (CSF). Culture has many limitations related to turnaround time and sensitivity, and also requires considerable infrastructure and costs ^7^. Therefore, the development of early point of care diagnosis for TBM is a priority. Recent studies have demonstrated that the next generation Xpert MTB/RIF Ultra is the most sensitive diagnostic test in HIV-positive adults ^7^. However, Xpert MTB/RIF Ultra is not a bedside test, and thus access to same day results remain a challenge in many settings ^7^.

Assays based on the detection of mycobacterial lipoarabinomannan (TB-LAM) antigen in urine have emerged as potential point-of-care tests for extra-pulmonary TB ^8^. There is evidence that urine TB-LAM may help to reduce mortality and predict poor outcomes ^9, 10^. The WHO recently added the TB-LAM assay onto its essential diagnostic list and recommended TB-LAM in hospitalised HIV positive adults with signs and symptoms of TB ^11, 12^. However, there are conflicting results about TB-LAM assay sensitivity for TBM diagnosis ^13^ in CSF. With reference to definite TBM, Cox *et al.* found a 75% sensitivity using CSF from the fourth ventricle in an autopsy cohort from 91 HIV-infected adults ^14^. However, Bahr *et al.* had no positive TB-LAM tests using lumbar CSF from 67 HIV patients with meningitis ^13^. In light of these results, and now that Xpert MTB/RIF Ultra is used instead of Xpert MTB/RIF, we aimed to further explore the utility of CSF TB-LAM test for the diagnosis of TBM among HIV-positive adults presenting with suspected meningitis.

## Methods

### Study setting and participants

Between April 2018 and June 2019, we assessed and performed diagnostic lumbar punctures on HIV-positive patients admitted to Mulago National Referral Hospital with suspected meningitis in Kampala, Uganda. Screening for TBM was performed cross-sectionally as part of the High Dose Rifampicin for Tuberculous Meningitis (RIFT) trial (ISRCTN registration number ISRCTN42218549, last updated 24/04/2018) ^15^. Therefore, we did not calculate a sample size for the current study but included all participants that fit the screening criteria for the RIFT trial ^15^. All included participants were HIV-infected adults (≥18 years old) who provided written informed consent by participant or surrogate, with a suspected diagnosis of TBM (meningitis symptoms, clinical signs of meningism). Demographic information and baseline characteristics for participants were collected through clinical reviews using customized meningitis screening case report forms approved by the relevant ethics committees (Mulago Hospital Research Ethics Committee, Uganda National Council of Science and Technology, and the University of Minnesota). Opening pressures for CSF were measured using a manometer, followed by standard microbiology analysis (CrAg, cell count, protein, glucose, lactate, culture).

### Diagnostic tests

In addition to standard microbiology analysis, CSF was tested with TB-LAM (Alere, Massachusetts, USA), and the test strip interpreted as per manufacturer’s instructions. Briefly, the protective foil cover was removed from each test and the strip labelled with the participant’s number. Two drops (or 60μL) of CSF were added to the sample pad. The test was then read after 25 minutes under standard indoor lighting conditions. The reference card was used in interpretation of the results by holding it alongside the patient window. For positive results, purple/gray bars appeared in both the control window and the patient window of the strip. For negative results, one purple/gray bar appeared in the control window of the strip and no bar appeared in the patient window of the strip. If there was no bar in the control window of the strip, the result was considered invalid and the test repeated. The strips were retained and cross checked by a second researcher to corroborate the finding.

CSF was also tested with Xpert MTB/Rif Ultra (Cepheid). Briefly, 2ml of sample reagent was added to 1ml of whole CSF and then left to stand at room temperature for 15 minutes. Then, 2ml of the sample mixture was transferred into the Xpert MTB/Rif Ultra cartridge and loaded into the Xpert machine. The test was run for 90 minutes and results from the assay indicate whether or not Mycobacteria TB (MTB) was detected in the sample. If MTB was detected, the results also stated whether resistance to rifampin was detected.

### Test analysis

Data were analyzed using STATA version 14 (STATA, College Station, Texas). The disease prevalence, sensitivity, specificity, positive predictive values, negative predictive values and test accuracy were estimated at 95% confidence interval (CI). The diagnostic performance of CSF TB-LAM was compared to positive CSF Xpert MTB/Rif Ultra (definite TBM) and a composite reference of probable or definite TBM according to the uniform case definition ^16^. Summaries were made in frequency & percentages for each baseline characteristic considered as a categorical, and medians (interquartile range) when each characteristic is considered as a continuous variable. For baseline variables with some missing data, we calculated the statistics using the available numbers.

### Ethical statement

Institutional review board approvals for the study and the associated screening process were obtained locally in Uganda (Mulago Hospital Research Ethics Committee, approval number MREC 1260); and from the London School of Hygiene and Tropical Medicine, UK (14388), University of Minnesota (1304M31361) and by the Uganda National Council of Science and Technology (HS136ES). Written informed consent for participation in the study and data publication was obtained from all participants or from their surrogates (e.g. family member or guardian) where the patient had altered mental status and did not have the capacity to provide consent.

## Results

Overall, 59 HIV-positive hospitalized participants with suspected meningitis underwent diagnostic lumbar punctures, of which 20% (12/59) had definite TBM, 9% (5/59) had probable TBM, 25% (15/59) had possible TBM, and 46% (27/59) had not-TBM ^17^. Of those with not-TBM (n=27), 10 had cryptococcosis. Women comprised 50% of participants with an overall median age for all participants of 33 years (interquartile range [IQR]: 28, 40). Only 29% of the participants were receiving antiretroviral therapy at diagnosis. Among participants reporting a headache (n=57), the median duration of headache was 14 days (IQR: 14, 24). The CSF opening pressures at baseline (n=45) had a median of 200 mmH _2_O (IQR: 120, 260). Overall, 55% (n=36) had an acellular CSF, whilst those with a CSF lymphocytic pleocytosis had a median CSF white blood cell of 160 cells/μL (IQR: 135, 268) ( Table 1). Only about 10% of the participants had cerebral imaging done as the CT scanner was dysfunctional for part of the study period. About twenty five percent of the patients had a positive TB-LAM while 20% had a positive urine MTB/Rif Ultra.

**Table 1.** Characteristics of the study population.

Baseline characteristics	Overall (N=59)		Definite TBM (n=12)		Probable TBM (n=5)		Possible TBM (n=15)		Not-TBM (n=27)	
	N *	Statistic	N *	Statistic	N *	Statistic	N *	Statistic	N *	Statistic
**Women, n (%)**	58	29 (50)	12	6 (50)	5	3 (60)	15	4 (26.7)	26	16 (61.5)
**Age in years, median (IQR)**	58	33 (28-40)	12	29 (28-33)	5	26 (24-34)	15	35 (32-43)	26	34 (26-46)
**On ART, n (%)**	47	29 (62)	11	7 (63.6)	2	2 (100)	11	3 (27.3)	23	17 (73.9)
**Headache, n (%)**	57	46 (81)	12	10 (83.3)	5	4 (80)	14	12 (85.7)	26	20 (76.9)
**Duration of headache,** **median (IQR) days**	45	14 (14-24)	10	17.5 (14-30)	4	14 (10.5-17.5)	12	17.5 (14-31.5)	19	14 (7-30)
**Glasgow Coma Scale score,** **mean (SD)**	55	13 (2.6)	12	12.5 (2.9)	5	11.8 (2.4)	14	12.7 (2.9)	24	14.3 (2.1)
**CSF CrAg positive, n (%)**	58	10 (17)	12	0 (0)	5	0 (0)	15	0 (0)	26	10 (38.4)
**CSF Opening Pressure,** **median (IQR) mmH2o**	45	200 (120-260)	7	180 (70-240)	3	260 (95-400)	13	190 (120-270)	22	215 (120-260)
**Acellular CSF, n (%)**	55	36 (55)	11	3 (27.3)	5	1 (20)	14	11 (78.6)	25	21 (84)
**CSF WBC in those with CSF** **WBC pleocytosis, median** **(IQR) cells/μL**	55	160 (135-268)	8	280 (162.5-575)	4	173 (130-237.5)	3	80 (35-160)	4	145 (87.5-210)
**CSF protein, median (IQR)** **mg/dL**	52	57 (28-141)	11	184 (107-316)	5	158 (147-215)	13	44 (35-72)	23	31 (22-61)
**CSF glucose, median (IQR)** **mg/dL**	32	65 (34-82)	7	44 (19.8-61)	3	90 (68-108)	8	86 (56.3-104)	14	61 (31-80)
**CSF lactate, median (IQR)** **mmol/L**	36	3.9 (2.2-9)	8	9.7 (8.2-11.2)	4	9.2 (6.3-11.1)	8	3.4 (2.3-8.1)	16	2.4 (1.9-3.8)
**Duration of hospitalization,** **median (IQR) days**	46	7 (4-14)	9	11 (9-14)	4	14.5 (10-16.5)	9	4 (4-14)	24	5 (2-12.5)
**Status at discharge** **Alive, n (%)** **Dead, n (%)** **Unknown, n (%)**	59	40 (68) 9 (15) 10 (17)	12	8 (66.7) 3 (25) 1 (8.3)	5	2 (40) 1 (20) 2 (40)	15	8 (53.3) 2 (13.3) 5 (33.3)	27	22 (81.5) 3 (11.1) 2 (7.4)

Data presented are percentages (%), medians and interquartile ranges (IQR). N= number of participants with data for each parameter. * Participants with data available. ART = antiretroviral therapy, CSF = cerebrospinal fluid, WBC = white blood cells.

With respect to the reference standard of definite TBM (positive CSF Xpert TB/Rif Ultra), the CSF TB-LAM assay had a sensitivity of 33% (4/12), specificity of 96% (45/47), positive predictive value (PPV) of 67% (4/6), and negative predictive value (NPV) of 85% (45/53). When compared to a composite reference of definite/probable TBM, the TB-LAM assay had a sensitivity of 24% (4/17), specificity of 95% (40/42), PPV of 67% (4/6), NPV of 76% (40/53) ( Table 2). There were two false positive tests with TB-LAM (3+ grade), without any CSF pleocytosis, normal protein, normal glucose, negative cryptococcal antigen, and normal CSF opening pressure. One patient was discharged alive without TB therapy. The second patient had a headache for 60 days at presentation, but they were lost to follow up (i.e. self-discharged) without an etiologic diagnosis. In-hospital mortality in CSF TB-LAM positive patients was 17% (1/6) compared to 0% (0/8) in those with definite TBM by Xpert MTB/Rif Ultra but negative LAM. About 17% of patients had unknown outcome. This was because the study population included patients screened for a clinical trial but only a minority were subsequently enrolled into the trial. We endeavoured to follow screen failures through to hospital discharge but this was not possible in all cases.

**Table 2.** Summary of diagnostic performance of cerebrospinal fluid mycobacterial lipoarabinomannan assay for tuberculous meningitis.

Reference standard	Disease prevalence	Sensitivity	Specificity	PPV	NPV	Test Accuracy
**Definite/probable** **TBM**	28.8% (17/59)	23.5% (4/17)	95.2% (40/42)	66.7% (4/6)	75.5% (40/53)	74.6% (44/59)
**95% CI**	17.8 to 42.1%	6.8 to 49.9%	83.8 to 99.4%	28.7 to 90.8%	70.1 to 80.2%	61.6 to 85%
**Definite TBM**	20.3% (12/59)	33.3% (4/12)	95.7% (45/47)	66.7% (4/6)	84.9% (45/53)	83.1% (49/59)
**95% CI**	10.9 to 32.8%	9.9 to 65.1%	85.5 to 99.5%	29.3 to 90.6%	78.9 to 89.4%	71 to 91.6%

Data presented are the percentage, numerator/denominator, and 95% confidence intervals (CI). Test Accuracy = overall probability that a patient will be correctly classified. PPV = Positive predictive value, NPV = negative predictive value, TBM = tuberculous meningitis.

## Conclusion

In conclusion, a rapid CSF point of care test for TBM is needed; however, this study demonstrated a poor diagnostic performance of the existing Alere TB-LAM on CSF among HIV-associated tuberculous meningitis. Our results corroborate the findings of a recent Zambian study which demonstrated 22% sensitivity for CSF LAM against a reference standard of TB culture ^18^. While the relatively modest sample size is a limitation, a larger sample size is unlikely to fundamentally alter the findings of sensitivity. One explanation could be that TB-LAM is likely not be found in sufficient quantities in lumbar CSF. TB culture was not used, which is also a limitation of the accuracy analysis. However, Xpert Ultra has a sensitivity that is greater than culture in our setting ^7^. The novel Fujifilm SILVAMP TB-LAM (FujiLAM) assay has been shown to have higher sensitivity in urine than the Alere TB-LAM and warrants evaluation for diagnosis of TB meningitis both in urine and CSF ^19^. 

## Data availability

### Underlying data

Figshare: CSFLAM_data set revised.xlsx. https://doi.org/10.6084/m9.figshare.9415853.v1 ^19^

Data are available under the terms of the Creative Commons Zero "No rights reserved" data waiver (CC0 1.0 Public domain dedication).

## References

[1] Jarvis JN Meintjes G Williams A: Adult meningitis in a setting of high HIV and TB prevalence: findings from 4961 suspected cases. *BMC Infect Dis.* 2010;10:67. 10.1186/1471-2334-10-67 20230635 PMC3161361

[2] Thao LTP Heemskerk AD Geskus RB: Prognostic Models for 9-Month Mortality in Tuberculous Meningitis. *Clin Infect Dis.* 2018;66(4):523–32. 10.1093/cid/cix849 29029055 PMC5850565

[3] Kalita J Misra U Ranjan P: Predictors of long-term neurological sequelae of tuberculous meningitis: a multivariate analysis. *Eur J Neurol.* 2007;14(1):33–7. 10.1111/j.1468-1331.2006.01534.x 17222110

[4] Saitoh A Pong A Waecker NJ Jr: Prediction of neurologic sequelae in childhood tuberculous meningitis: a review of 20 cases and proposal of a novel scoring system. *Pediatr Infect Dis J.* 2005;24(3):207–12. 10.1097/01.inf.0000154321.61866.2d 15750455

[5] Heemskerk AD Bang ND Thwaites GE: Therapy for Tuberculous Meningitis. *N Engl J Med.* 2016;374(22):2188–9. 10.1056/NEJMc1602291 27248634

[6] Ndyetukira JF Kwizera R Kugonza F: The conundrum of clinical trials and standard of care in sub-Saharan Africa – the research nurse perspective. *J Res Nurs.* 2019 10.1177/1744987118824625 PMC7932327 34394589

[7] Bahr NC Nuwagira E Evans EE: Diagnostic accuracy of Xpert MTB/RIF Ultra for tuberculous meningitis in HIV-infected adults: a prospective cohort study. *Lancet Infect Dis.* 2018;18(1):68–75. 10.1016/S1473-3099(17)30474-7 28919338 PMC5739874

[8] Songkhla MN Tantipong H Tongsai S: Lateral Flow Urine Lipoarabinomannan Assay for Diagnosis of Active Tuberculosis in Adults With Human Immunodeficiency Virus Infection: A Prospective Cohort Study. *Open Forum Infect Dis.*Oxford University Press US.2019;6(4): ofz132. 10.1093/ofid/ofz132 31024973 PMC6475588

[9] Gupta-Wright A Corbett EL Wilson D: Risk score for predicting mortality including urine lipoarabinomannan detection in hospital inpatients with HIV-associated tuberculosis in sub-Saharan Africa: Derivation and external validation cohort study. *PLoS Med.* 2019;16(4):e1002776. 10.1371/journal.pmed.1002776 30951533 PMC6450614

[10] Gupta-Wright A Corbett EL van Oosterhout JJ: Rapid urine-based screening for tuberculosis in HIV-positive patients admitted to hospital in Africa (STAMP): a pragmatic, multicentre, parallel-group, double-blind, randomised controlled trial. *Lancet.* 2018;392(10144):292–301. 10.1016/S0140-6736(18)31267-4 30032978 PMC6078909

[11] WHO: The use of lateral flow urine lipoarabinomannan assay (LF-LAM) for the diagnosis and screening of active tuberculosis in people living with HIV. Geneva, Switzerland.2015 Reference Source

[12] Bongomin F Govender NP Chakrabarti A: Essential *in vitro* diagnostics for advanced HIV and serious fungal diseases: international experts' consensus recommendations. *Eur J Clin Microbiol Infect Dis.* 2019;38(9):1581–1584. 10.1007/s10096-019-03600-4 31175479

[13] Bahr NC Tugume L Boulware DR: A Word of Caution in Considering the Use of the Lipoarabinomannan Lateral Flow Assay on Cerebrospinal Fluid for Detection of Tuberculous Meningitis. *J Clin Microbiol.* 2016;54(1):241–2. 10.1128/JCM.02753-15 26719583 PMC4702762

[14] Cox JA Lukande RL Kalungi S: Accuracy of Lipoarabinomannan and Xpert MTB/RIF Testing in Cerebrospinal Fluid To Diagnose Tuberculous Meningitis in an Autopsy Cohort of HIV-Infected Adults. *J Clin Microbiol.* 2015;53(8):2667–73. 10.1128/JCM.00624-15 26063865 PMC4508395

[15] Cresswell FV Ssebambulidde K Grint D: High dose oral and intravenous rifampicin for improved survival from adult tuberculous meningitis: a phase II open-label randomised controlled trial (the RifT study) [version 1; peer review: 2 approved]. *Wellcome Open Res.* 2018;3:83. 10.12688/wellcomeopenres.14691.1 30175245 PMC6113880

[16] Marais S Thwaites G Schoeman JF: Tuberculous meningitis: a uniform case definition for use in clinical research. *Lancet Infect Dis.* 2010;10(11):803–12. 10.1016/S1473-3099(10)70138-9 20822958

[17] Kwizera R: CSFLAM_data set revised. xlsx. figshare.com.2019 10.6084/m9.figshare.9415853.v1

[18] Siddiqi OK Birbeck GL Ghebremichael M: Prospective Cohort Study on Performance of Cerebrospinal Fluid (CSF) Xpert MTB/RIF, CSF Lipoarabinomannan (LAM) Lateral Flow Assay (LFA), and Urine LAM LFA for Diagnosis of Tuberculous Meningitis in Zambia. *J Clin Microbiol.* 2019;57(8): pii: e00652-19. 10.1128/JCM.00652-19 31189584 PMC6663887

[19] Broger T Sossen B du Toit E: Novel lipoarabinomannan point-of-care tuberculosis test for people with HIV: a diagnostic accuracy study. *Lancet Infect Dis.* 2019;19(8):852–61. 10.1016/S1473-3099(19)30001-5 31155318 PMC6656794

