# Supplementary material for: Performance of Lipoarabinomannan Assay using Cerebrospinal fluid for the diagnosis of Tuberculous meningitis among HIV patients
Source: Wellcome Open Res. 2019 Sep 30;4:123. Originally published 2019 Aug 19. [Version 2] doi: 10.12688/wellcomeopenres.15389.2 (PMC6749932; doi:10.12688/wellcomeopenres.15389.2)
Supplement: Peer review history

# Peer review: Reviewer response for version 2

**DOI:** 10.21956/wellcomeopenres.16956.r36606

**Published:** 2019-10-02

**Authors:** Maryline Bonnet

**Affiliations:** 1 Institute of Research for Development (IRD), Montpellier, France

I confirm that I have read this submission and believe that I have an appropriate level of expertise to confirm that it is of an acceptable scientific standard.

# Peer review: Reviewer response for version 1

**DOI:** 10.21956/wellcomeopenres.16813.r36425

**Published:** 2019-09-16

**Authors:** Vinod Patel

**Affiliations:** 1 Department of Neurology, Nelson R. Mandela School of Medicine, University of KwaZulu-Natal (UKZN), Durban, South Africa

The article is well written with no major flaws.

 Regarding the non-TBM category, although 10 patient had cryptococcal meningitis, detail regarding the non-TBM diagnoses is important as this is a control group and a patient without a clinical meningitis may allow for better specificity. I note that some CSF’s were acellular, what were these diagnoses?

 Please provide detail (a table with confirmatory findings for TB such as CXR, abdominal ultrasound, scan findings, CSF findings etc.) regarding the aspects considered to arrive at a diagnosis of probable and possible TBM. A similar consideration for possible TBM. This would add clarity on the reliability of the test outcomes.

I confirm that I have read this submission and believe that I have an appropriate level of expertise to confirm that it is of an acceptable scientific standard.

# Response

**Published:** 2019-09-26

**Authors:** Richard Kwizera

**Affiliations:** Makerere University, Uganda

The article is well written with no major flaws.

**Response:** Thank you.

Regarding the non-TBM category, although 10 patient had cryptococcal meningitis, detail regarding the non-TBM diagnoses is important as this is a control group and a patient without a clinical meningitis may allow for better specificity. I note that some CSF’s were acellular, what were these diagnoses? Please provide detail (a table with confirmatory findings for TB such as CXR, abdominal ultrasound, scan findings, CSF findings etc.) regarding the aspects considered to arrive at a diagnosis of probable and possible TBM. A similar consideration for possible TBM. This would add clarity on the reliability of the test outcomes.

**Response:** A cellular CSF is common in this population with advanced HIV disease even in the setting of a confirmed TB or CM infection (Cresswell, Int J Infect Dis 2018). The not-TBM group scored <6 points on the uniform case definition (n=16) or had a confirmed alternative diagnosis (n=10 with CM).

# Peer review: Reviewer response for version 1

**DOI:** 10.21956/wellcomeopenres.16813.r36338

**Published:** 2019-09-09

**Authors:** Maryline Bonnet

**Affiliations:** 1 Institute of Research for Development (IRD), Montpellier, France

This manuscript reports the results of a nested study in a large clinical trial, evaluating the accuracy of the CSF LAM for diagnosis of tuberculosis meningitis using both definite TB and composite reference of probable and definite TB based on standard case definitions. The study reports low sensitivity and high specificity and concludes on the modest role of the CSF LAM for diagnosis of TB meningitis. These results are important and the manuscript is well written.

 No major comments.

 Minor comments:

 In the introduction, I suggest the authors adding the recent publication by Siddiqi *et al*.(2019) ^1^ in their references.

 In Results, I would suggest the authors to present the characteristics of patients with diagnosis of probable TB using the uniform case definitions. I am surprised by the low proportion of probable TB as compared to definite TB. It would be interesting to know the score of patients with probable TB using the uniform case definition criteria. Were cerebral imaging criteria used? It would be also interesting to know the proportion of patients that fit the score of possible TB meningitis using the uniform case definitions. It is indeed important to know the proportion of possible TB meningitis cases based on the uniform cases definitions that were finally classified as non TB meningitis for the accuracy analysis.

 It would be interesting to know the proportion of patients with TB positive results from another specimen than CSF, which is also an important criteria for diagnosis of probable TB meningitis. One option could be to present the patients’ characteristics by definite TB, probable TB and others in Table 1.

 How do the authors explain that 17% of patients had unknown outcome of death or alive at discharge? It is quite high in a context of a nested study in a clinical trial.

 TB culture was not used, which is a limitation of the accuracy analysis. However, Xpert Ultra has a sensitivity that is very close to culture. This could be mentioned as a limitation.

 It would be also very interesting to have the results of the urine LAM if used. In the study by Siddiqui *et al.* the urine LAM had higher sensitivity than the CSF LAM in patients with presumptive TB meningitis.

 In the conclusion, the authors could mention the Fuji LAM that is a new LAM POC test that has higher sensitivity in urine than the determine LAM POC and should also be evaluated for diagnosis of TB meningitis both in urine and CSF.

I confirm that I have read this submission and believe that I have an appropriate level of expertise to confirm that it is of an acceptable scientific standard.

## References

[1] : Prospective Cohort Study on Performance of Cerebrospinal Fluid (CSF) Xpert MTB/RIF, CSF Lipoarabinomannan (LAM) Lateral Flow Assay (LFA), and Urine LAM LFA for Diagnosis of Tuberculous Meningitis in Zambia. *J Clin Microbiol*.2019;57(8) : 10.1128/JCM.00652-19 10.1128/JCM.00652-19 31189584PMC6663887

# Response

**Published:** 2019-09-26

**Authors:** Richard Kwizera

**Affiliations:** Makerere University, Uganda

This manuscript reports the results of a nested study in a large clinical trial, evaluating the accuracy of the CSF LAM for diagnosis of tuberculosis meningitis using both definite TB and composite reference of probable and definite TB based on standard case definitions. The study reports low sensitivity and high specificity and concludes on the modest role of the CSF LAM for diagnosis of TB meningitis. These results are important and the manuscript is well written.

**Response:** Thank you

No major comments.

Minor comments:

**Comment:** In the introduction, I suggest the authors adding the recent publication by Siddiqi *et al*.(2019) ^1^ in their references.

**Response:** We have now added this suggested reference in the discussion section.

Comment: In Results, I would suggest the authors to present the characteristics of patients with diagnosis of probable TB using the uniform case definitions.

**Response:** Table 1 has been modified to present the characteristics of patients with diagnosis of definite, probable, possible and not-TBM.

Comment: I am surprised by the low proportion of probable TB as compared to definite TB.

**Response:** This is likely to be because we used Ultra and not culture or Xpert as the reference standard. We have found Ultra to be better at confirming TBM in our population (Bahr, Lancet ID, 2018)

Comment: Were cerebral imaging criteria used?

**Response**: Only about 10% (n/N) of the participants had cerebral imaging done as the CT scanner was dysfunctional for part of the study period.

Comment: It would be also interesting to know the proportion of patients that fit the score of possible TB meningitis using the uniform case definitions.

**Response:** These have been added in Table 1.

Comment: It would be interesting to know the proportion of patients with TB positive results from another specimen than CSF, which is also an important criteria for diagnosis of probable TB meningitis.

**Response:** 25% of the patients were positive with urine LAM while 20% were positive with urine Ultra.

Comment: One option could be to present the patients’ characteristics by definite TB, probable TB and others in Table 1.

**Response**: Thank you for this suggestion. This has been done in the modified Table 1.

Comment: How do the authors explain that 17% of patients had unknown outcome of death or alive at discharge? It is quite high in a context of a nested study in a clinical trial.

**Response:** This study population included patients screened for a clinical trial but only a minority were subsequently enrolled into the trial. We endeavoured to follow screen failures through to hospital discharge but this was not possible in all cases.

Comment: TB culture was not used, which is a limitation of the accuracy analysis. However, Xpert Ultra has a sensitivity that is very close to culture. This could be mentioned as a limitation.

**Response:** We have found Ultra to be more sensitive than culture (70% versus 43% against definite/probable) for the diagnosis of TBM in our population (Bahr, Lancet ID, 2018). However we agree addition to culture would have been positive so this has been added to the limitations.

Comment: It would be also very interesting to have the results of the urine LAM if used. In the study by Siddiqui *et al*. the urine LAM had higher sensitivity than the CSF LAM in patients with presumptive TB meningitis.

**Response:** 25% of the patients were positive with urine TB-LAM LFA (Alere). The details of the urine TB diagnostics are being analysed currently and will be presented separately.

Comment: In the conclusion, the authors could mention the Fuji LAM that is a new LAM POC test that has higher sensitivity in urine than the determine LAM POC and should also be evaluated for diagnosis of TB meningitis both in urine and CSF.

**Response:** Thank you for this suggestion. This has been added in the conclusion.
